# Endothelium-derived extracellular vesicles promote splenic monocyte mobilization in myocardial infarction

**DOI:** 10.1172/jci.insight.93344

**Published:** 2017-09-07

**Authors:** Naveed Akbar, Janet E. Digby, Thomas J. Cahill, Abhijeet N. Tavare, Alastair L. Corbin, Sushant Saluja, Sam Dawkins, Laurienne Edgar, Nadiia Rawlings, Klemen Ziberna, Eileen McNeill, Oxford Acute Myocardial Infarction (OxAMI) Study, Errin Johnson, Alaa A. Aljabali, Rebecca A. Dragovic, Mala Rohling, T. Grant Belgard, Irina A. Udalova, David R. Greaves, Keith M. Channon, Paul R. Riley, Daniel C. Anthony, Robin P. Choudhury

**Affiliations:** 1 Division of Cardiovascular Medicine, Radcliffe Department of Medicine, and; 2 Kennedy Institute of Rheumatology, University of Oxford, Oxford, United Kingdom.; 3 The OxAMI Study is detailed in the Supplemental Acknowledgments.; 4 Sir William Dunn School of Pathology,; 5 Nuffield Department of Obstetrics and Gynaecology, and; 6 Department of Physiology, Anatomy and Genetics, University of Oxford, Oxford, United Kingdom.; 7 Verge Genomics, San Francisco, California, USA.; 8 Experimental Neuropathology, Department of Pharmacology, and; 9 Acute Vascular Imaging Centre, Radcliffe Department of Medicine, University of Oxford, Oxford, United Kingdom.

**Keywords:** Cardiology, Vascular Biology, Cell migration/adhesion, Monocytes, endothelial cells

## Abstract

Transcriptionally activated monocytes are recruited to the heart after acute myocardial infarction (AMI). After AMI in mice and humans, the number of extracellular vesicles (EVs) increased acutely. In humans, EV number correlated closely with the extent of myocardial injury. We hypothesized that EVs mediate splenic monocyte mobilization and program transcription following AMI. Some plasma EVs bear endothelial cell (EC) integrins, and both proinflammatory stimulation of ECs and AMI significantly increased VCAM-1–positive EV release. Injected EC-EVs localized to the spleen and interacted with, and mobilized, splenic monocytes in otherwise naive, healthy animals. Analysis of human plasma EV-associated miRNA showed 12 markedly enriched miRNAs after AMI; functional enrichment analyses identified 1,869 putative mRNA targets, which regulate relevant cellular functions (e.g., proliferation and cell movement). Furthermore, gene ontology termed positive chemotaxis as the most enriched pathway for the miRNA-mRNA targets. Among the identified EV miRNAs, EC-associated miRNA-126-3p and -5p were highly regulated after AMI. miRNA-126-3p and -5p regulate cell adhesion– and chemotaxis-associated genes, including the negative regulator of cell motility, plexin-B2. EC-EV exposure significantly downregulated plexin-B2 mRNA in monocytes and upregulated motility integrin ITGB2. These findings identify EVs as a possible novel signaling pathway by linking ischemic myocardium with monocyte mobilization and transcriptional activation following AMI.

## Abstract 2

Acute myocardial infarction elevates circulating endothelial cell–derived extracellular vesicles, which can mobilize monocytes from the spleen and alter transcription.

## Introduction

Tissue injury sustained after acute myocardial infarction (AMI) results in local necrosis and inflammation. AMI induces a peripheral monocytosis in both humans and mice, which is positively correlated with the extent of myocardial injury (1). Recent pivotal studies have shown that activation of the innate immune system in AMI sequentially mediates aspects of both injury and repair (1–4). Importantly, early-phase monocytes appear to act as mediators of injury, since attenuating the response of inflammatory monocytes with siRNA (5, 6), angiotensin-converting enzyme inhibitors (7, 8) or splenectomy (2, 7) significantly reduced infarct size in experimental models. Therefore, a better understanding of the processes involved in monocyte activation and recruitment to the damaged myocardium might allow therapeutic intervention to alter favorably the milieu for repair and regeneration and to extend the very short time window of efficacy for current treatments of AMI.

Following AMI, monocytes are mobilized in large numbers from the spleen, activated, and recruited to the damaged myocardium (1, 2). We have recently shown that, in both mice and humans, AMI is associated with distinct patterns of gene expression in monocytes, which are conserved between species (1). Significantly these altered patterns of gene expression are apparent in circulating monocytes *before* their recruitment to the sites of injury. Stimuli for monocyte mobilization include β-adrenergic signals (9) and soluble factors, e.g., angiotensin II (7, 10). However, the mechanism by which the injured myocardium signals both monocyte mobilization and transcriptional activation from the splenic reserve remains unknown.

Extracellular vesicles (EVs) are effective mediators of cell-to-cell communication, both locally and remotely, between different organs (11), under normal and pathological conditions (12, 13). EVs are membrane-encapsulated vesicles that are actively released from cells. EVs with the size range of 50–200 nm in diameter contain a mixed population of exosomes, which are formed by inward budding of multivesicular bodies. They bear proteins and nucleic acids derived from the cell of origin, including tetraspanins and adhesion molecules, such as integrins (12, 14). These characteristics enable interaction with receptors on target cells to promote signaling pathways that modulate the function of recipient cells (15).

microRNA (miRNA), other small noncoding RNA sequences, and mRNA (16–21) carried in EVs are protected from degradation, as they are conveyed in the blood and can alter gene expression and cellular phenotype in recipient cells (18, 22–24). Using a multimodal imaging reporter, clearance of EVs from the blood has been shown to be rapid and tissue specific, predominantly to the spleen (25). Furthermore, EVs are taken up by monocytes and macrophages in the spleen and liver, and, in clodronate liposome macrophage-depleted mice, EV clearance is abrogated (26). EVs are readily released from damaged/stimulated endothelial cells (ECs) (27–29). Given their composition and mode of clearance, we reasoned that EVs may signal from injured ECs of the myocardial microvasculature to the spleen, contributing to the mobilization and programming of monocytes that occurs in AMI (1, 2). Accordingly, we aimed to determine the changes in EVs following AMI in mice and in humans; to define their cell type of origin; and to characterize changes in their composition (miRNA and protein). Informed by the findings, we further tested the effects of EVs on monocyte gene expression and function.

## Results

### Circulating plasma EV concentrations increase with myocardial ischemic injury.

**Figure 1:**
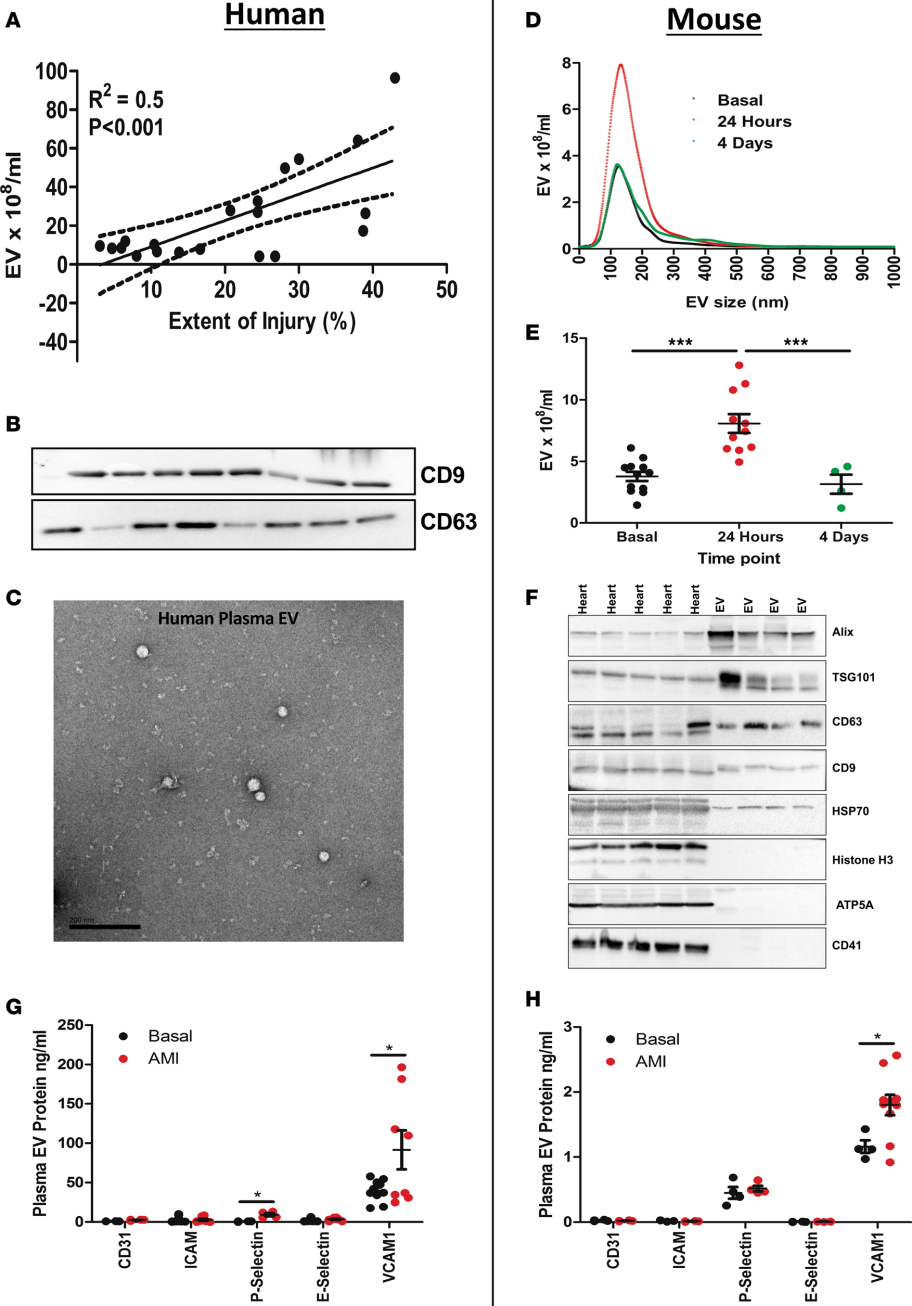
Circulating plasma EV concentration increase with myocardial ischemic injury. (**A**) Pearson correlation between the number of plasma extracellular vesicles (EVs, 10^8^/ml) and the degree of ischemic injury in humans (*n* = 22) in acute myocardial infarction (AMI). (**B**) Western blot analysis of human plasma EVs for CD9 and CD63. (**C**) TEM of human plasma EVs. Scale bar: 200 nm. (**D** and **E**) Nanoparticle tracking analysis of plasma EVs from wild-type mice subjected to experimental AMI (24 hours) (*n* = 11–12). (**F**) Western blot analysis of mouse plasma EVs for ALIX, TSG101, CD63, CD9, Hsp70, ATP5A, histone H3, and CD41. (**G**) Human (*n* = 8–24) and (**H**) mouse (*n* = 8–13) plasma EV surface markers, CD31, ICAM-1, P selectin, E selectin, and VCAM-1. Values are group mean ± SEM. One-way ANOVA with post-hoc Bonferroni correction or unpaired *t* test. *P < 0.05, ***P < 0.01.

We established the relationship between the number and/or size distribution of plasma EVs and acute myocardial ischemic injury in patients with ST-segment elevation AMI. We obtained blood at the time of presentation with AMI and found that the number of EVs in peripheral blood after AMI positively correlated (R^2^ = 0.52, *P* < 0.01) with the extent of acute ischemic injury, determined by edema quantification on T2-weighted MRI, within 48 hours of presentation (Figure 1A). The number of total circulating plasma EVs was significantly greater at the time of presentation than at follow-up at 6 months after AMI (2.3-fold; *P* < 0.01) (Supplemental Figure 1, A and B; supplemental material available online with this article; https://doi.org/10.1172/jci.insight.93344DS1).

In a mouse model of AMI, total circulating plasma EVs significantly increased (2.1-fold; *P* < 0.001) acutely (24 hours) after AMI and subsided in the succeeding days (*P* < 0.001) (Figure 1, D and E). EV size distribution was similar across all time points (modal EV size: 129 ± 7 nm; 24 hours after AMI, 126 ± 5 nm; 4 days after MI, 129 ± 19 nm), with a specific increase in exosome number (EV <150 nm) after AMI (Figure 1, D and E, and Supplemental Figure 1, A and B).

The integrity of EV isolation from plasma was confirmed by immunoblotting for recognized EV protein markers: ALIX, the tumor susceptibility gene 101 (TSG101), tetraspanins CD63/CD9, and heat shock protein 70 (Hsp70) were identified in the plasma EV preparations (Figure 1, B and F). The purity of isolated plasma EV was confirmed by the absence of “whole-cell” components, such as the nuclear protein histone H3 and the mitochondrial protein ATP5A. Since, the isolation of plasma EV can be subject to contamination by circulating platelets, we used platelet marker CD41 to confirm their absence (Figure 1F). In addition, isolated plasma EV preparations were imaged using transmission electron microscopy (TEM) to confirm their typical morphology (Figure 1C).

### Circulating plasma EVs show EC origin.

AMI is associated with endothelial activation and recruitment of inflammatory cells. Given their direct adjacency to the blood, ECs represent a potentially efficient source of EVs to signal to remote organs. Therefore, to gauge the contribution of ECs to the change in the total EV population, we measured EC markers in human and mouse plasma EVs following AMI. In human plasma-derived EVs, there were significant increases in P selectin (CD62p) (13.7-fold, *P* < 0.05; *n* = 8) and VCAM-1, (2.3-fold, *P* < 0.001) compared with control stable atherosclerosis patients (Figure 1G). Similarly, EVs isolated from mouse plasma displayed PECAM-1, ICAM-1, VCAM-1, E selectin, and P selectin surface markers, further suggesting EC origin of some plasma EVs (Figure 1H). The plasma population of EVs is heterogeneous and reflects multiple potential cell types of origin. To ascertain the proportion of plasma EVs with likely EC origin, we used magnetic beads bearing anti-VCAM-1 antibodies to extract EVs expressing VCAM-1. Through quantification of plasma EVs, we found a greater number of EC-related glycoprotein VCAM-1–positive EVs after AMI (VCAM-1^+^ EV: uninjured 13% vs. AMI 33%, *P* < 0.01).

### ECs increase EV release after proinflammatory cytokine stimulation.

**Figure 2:**
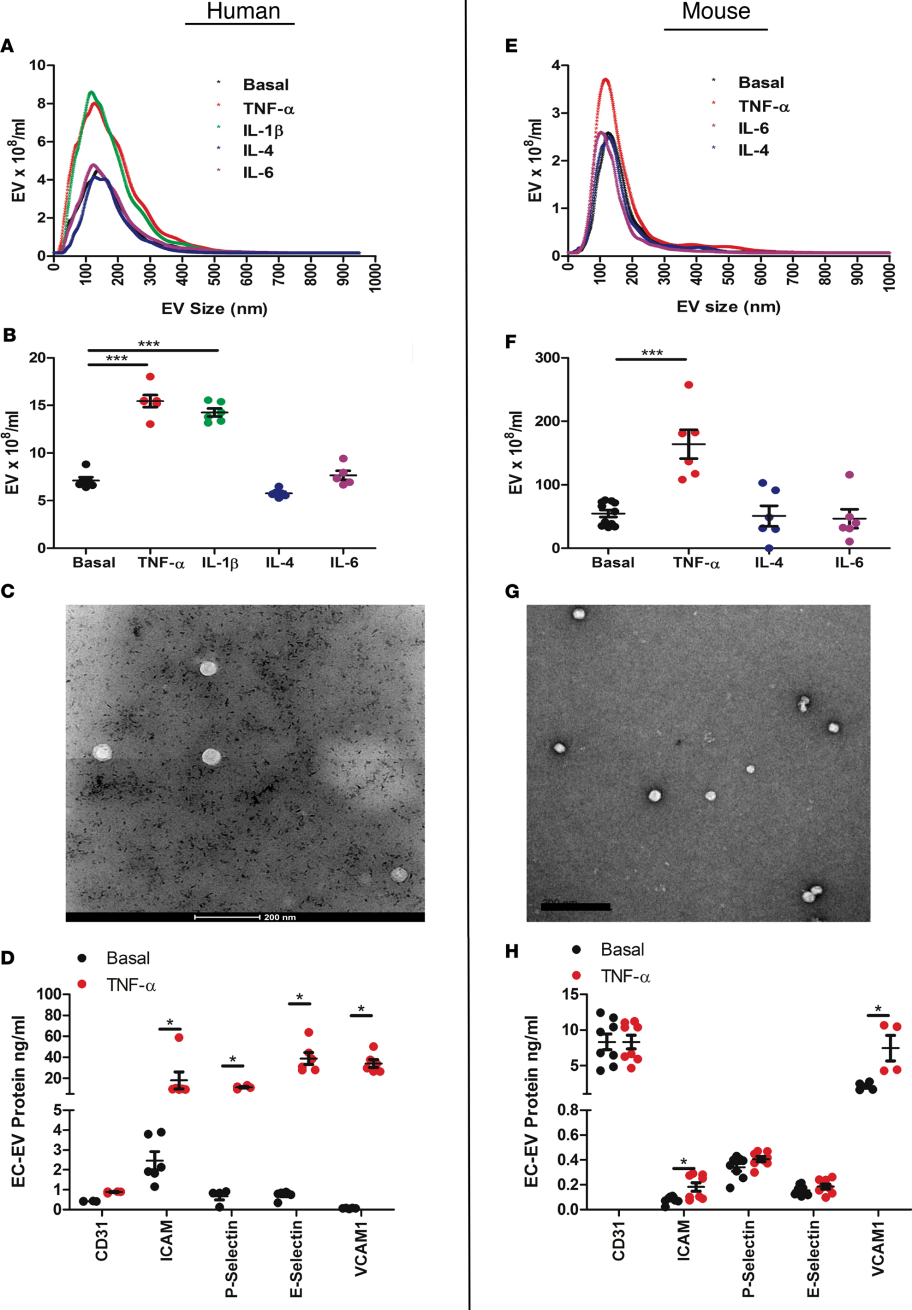
Endothelial cells increase EV release after proinflammatory cytokine stimulation. (**A** and **B**) Nanoparticle tracking analysis (NTA) of human umbilical vein endothelial cell–derived (HUVEC-derived) extracellular vesicles (EVs) released under basal and inflammatory stimulated conditions: TNF-α, IL-1β, IL-4, and IL-6 (*n* = 5–6). (**C**) TEM of HUVEC EVs. Scale bar: 200 nm. (**D**) HUVEC EV surface markers ICAM-1, VCAM-1, P selectin, and E selectin under basal and inflammatory conditions (*n* = 4–16 per group). (**E** and **F**) NTA of sEND.1 EVs released under basal and inflammatory-stimulated conditions: TNF-α, IL-4, and IL-6 (*n*= 5–11). (**G**) TEM of sEND.1 EVs. Scale bar: 200 nm. (**H**) s.END1 EV surface markers ICAM-1, VCAM-1, P selectin, and E selectin under basal and inflammatory conditions (*n* = 4–8 per group). Values are group mean ± SEM. (**A**, **B**, **E**, and **F**) One-way ANOVA with post-hoc Bonferroni correction or (**D** and **H**) unpaired *t* test. *P < 0.05, ***P < 0.001.

To characterize more closely EVs of pure EC origin, we used an in vitro model of EC activation. Proinflammatory cytokines, including TNF-α and IL-1β, induce EC activation with upregulation of adhesion molecules (VCAM-1 and ICAM-1), and we hypothesized that they may also promote EV release. Compared with basal conditions, treatment of human umbilical vein ECs (HUVECs) with TNF-α (10 ng/ml) or IL-1β (10 ng/ml) increased EV production by 2.2-fold and 2.0-fold, respectively (*P* < 0.001 for each) (Figure 2, A and B). Conversely, IL-4 and IL-6 had no effect on EV release from ECs (Figure 2, A and B). HUVEC-derived EVs displayed typical morphology (Figure 2C), and TNF-α–stimulated HUVEC-derived EVs showed significant enrichment for EC activation markers ICAM-1, P selectin, E selectin, and VCAM-1 when compared with basal, unstimulated HUVEC EVs (Figure 2D).

These data suggest that proinflammatory cytokines selectively increase EV production, which are also enriched for EC activation markers, compared with basal conditions, but without affecting modal size distribution (Figure 2A). Similarly, the mouse microvasculature endothelioma s.END1 cell line stimulated with TNF-α significantly increased EV number (Figure 2, E and F), maintaining characteristic morphology (Figure 2G) and enrichment for ICAM-1 and VCAM-1 (Figure 2H). We further confirmed these findings in primary cardiac mouse ECs (Supplemental Figure 1, C and D) and ascertained that ECs, but not primary cardiomyocytes, increase EV release after inflammatory stimulation (Supplemental Figure 1, E and F).

To confirm the origin of EC-derived EVs (EC-EVs) in our cell culture preparations, we prepared sham EV isolations using EV-depleted medium that was not exposed to cells for EV isolation. Sham EC-EV preparations were EV null, as shown by nanoparticle tracking analysis (Supplemental Figure 2, A and B, and Supplemental Figure 2, F and G); had lower protein concentrations (Supplemental Figure 2, C and H); had fewer protein bands on a Coomassie gel (Supplemental Figure 2, D and I); and were void of EV-associated proteins by Western blot (ALIX, TSG101, and CD9) (Supplemental Figure 2, E and J).

### EC-EVs are taken up by monocytes.

**Figure 3:**
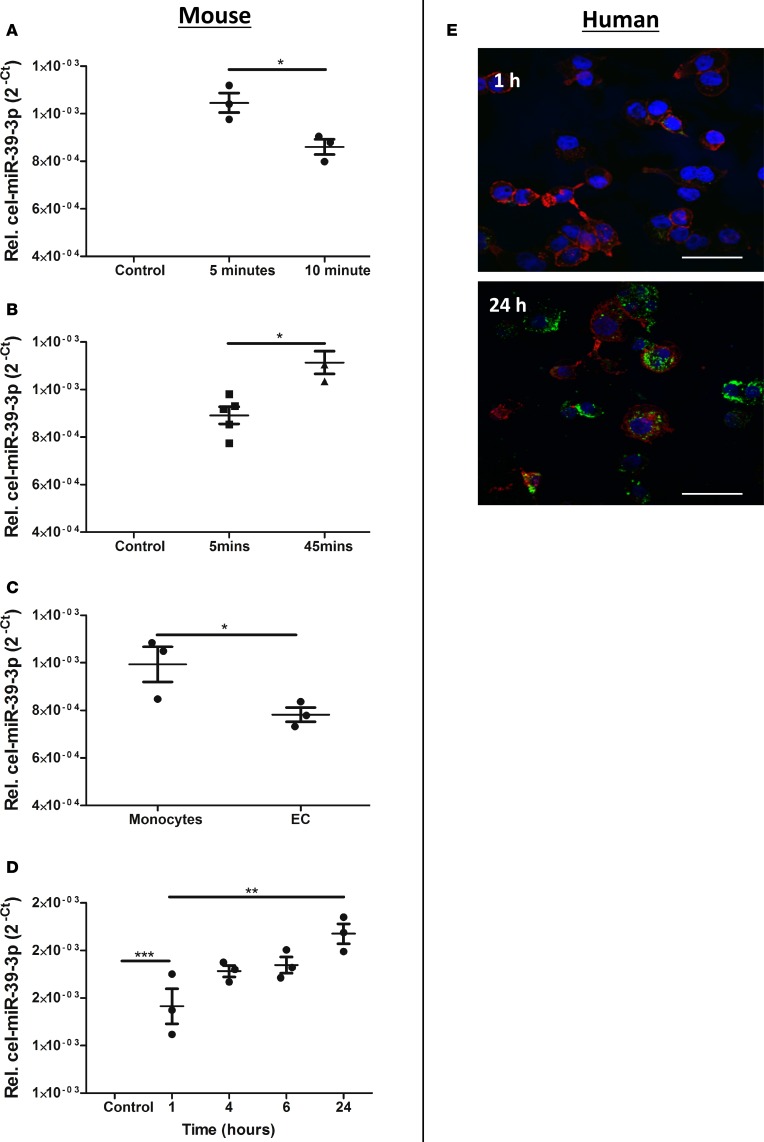
EC-EVs are taken up by monocytes. (**A**) Tail vein–injected endothelial cell–derived extracellular vesicles (EC-EVs) transfected with cel-miR39 in mouse blood, (**B**) spleen, and (**C**) splenic monocytes (*n* = 3–5). (**D**) EC-EVs transfected with cel-miR39 uptake by RAW264.7 cells. (**E**) PKH67-labeled (green) HUVEC-derived EVs accumulate THP-1 monocyte-derived macrophages (scale bar: 100 μm) (red: F-actin [Phalloidin], nucleus [DAPI: blue]). Group values are 2^–Ct^ (group mean ± SEM). Unpaired 2-tailed *t* test. *P < 0.05, **P < 0.01.

Previous studies of EV clearance have shown rapid accumulation in the spleen (26, 30). In order to determine the splenic uptake of EC-derived EVs, we generated EV in cell cultures (s.END1) that were then labeled with the *C*. *elegans* miRNA, cel-miR39-3p, to allow quantitative tracing of EC-EVs over time by qPCR. After a bolus injection through the tail vein in mice, EC-EVs left the systemic blood pool (Figure 3A) and accumulated in the spleen (Figure 3B), accumulating more preferentially in splenic monocytes than in splenic ECs (Figure 3C). To confirm the interaction between EC-derived EVs and monocytes, we utilized RAW264.7 cells and THP.1-differentiated macrophages in vitro and found that cel-miR39-3p–labeled s.END1 EVs (Figure 3D) and PKH67-labeled HUVEC-derived EVs (Figure 3E) accumulated in RAW264.7- and THP.1-derived macrophages, respectively, over time.

### EV miRNA content changes after AMI.

Since EVs carry miRNA, we next undertook a screen of a panel of 752 miRNAs in EVs that had been isolated from the plasma of 20 patients who experienced AMI. Of these, we obtained miRNA for 19 (95%) patients that passed the quality control measures (Table 1). EVs from patients experiencing AMI contained a relatively small number of miRNA when compared with whole plasma (data not shown), suggesting both compartmentalization and enrichment of specific miRNA in EVs. We then designated a validation panel of 90 miRNAs, which were present in EVs from the initial discovery panel and tested these in a further 23 patients. We found substantial changes in 12 EV miRNAs (Table 2) following an elastic linear regression model.

### Identification of miRNA-mRNA targets in EVs.

mRNA target prediction was performed using Ingenuity Pathways Analysis (IPA) for the differentially enriched miRNA in plasma EVs following AMI. The analysis revealed 2,371 putative mRNA targets by 10 miRNAs from TarBase, TargetScan Human, and miRecords. miRNA-mRNA targets were subjected to core analysis in IPA, which suggested putative functions in the regulation of cellular growth and proliferation, cell death and survival, and cellular movement. Furthermore IPA-identified miRNA-mRNA targets were imputed into gene ontology (GO) analysis to define GO biological process terms. 86 genes appeared in the mRNA target list more than once and 1,869 unique mapped gene IDs were compared with a reference list of the human genome and ranked by fold enrichment. The most enriched GO biological process term was for positive regulation of positive chemotaxis (GO: 0050927).

EC-associated miRNA-126-3p and miRNA-126-5p (31, 32) were highly regulated in EVs following AMI (Table 2). Functional enrichment analyses using GO biological terms suggested a role for miRNA-126-3p and -5p in the regulation of genes associated with lymphocyte differentiation, positive regulation of cell adhesion, and regulation of cell motility, including for the negative regulator of cell motility plexin-B2 (33).

**Figure 4:**
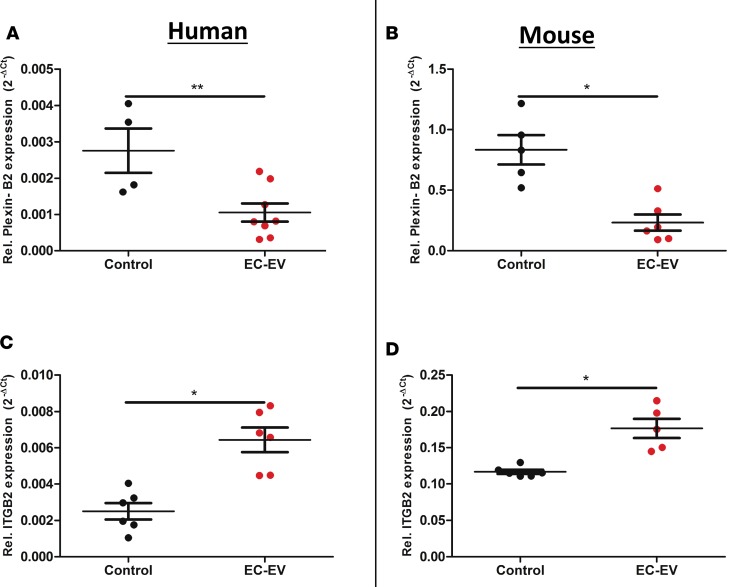
EC-EVs alter mRNA expression in monocytes. (**A**) Plexin-B2 mRNA in THP-1 cells exposed to HUVEC EVs and (**B**) in RAW264.7 cell exposed to s.END1 EVs. (**C**) THP-1 cells and (**D**) RAW264.7 cells show altered relative mRNA expression for ITGB2 after EC-EV exposure. Values are normalized to cyclophilin. Group mean ± SEM 2^-ΔCt^. Unpaired *t* test (*n* = 4–8). *P < 0.05, **P < 0.01.

THP-1 and RAW264.7 cells exposed to “inflammatory” EC-EVs significantly downregulated plexin-B2 (*P* < 0.01) (Figure 4, A and B), confirming the predictions from functional enrichment analyses and further suggesting that EVs may play a role in enhanced cellular motility. To better understand the transcriptional changes in genes associated with cellular motility in monocytes exposed to EC-EVs, we undertook a “chemotaxis mRNA array” and analyzed THP-1 cells that were exposed to EVs derived from TNF-α–stimulated HUVECs. Exposure to EC-EVs substantially altered a number of chemotaxis-associated genes (Table 3), including integrin subunit α 4 (ITGA4) and integrin subunit β 2 (ITGB2), which are key integrin chain molecules used in leucocyte adhesion and cellular motility for tissue recruitment (34, 35). HUVEC-derived EVs significantly upregulated ITGB2 in THP-1 monocytes (3.5-fold, *P* < 0.01) and s.END1-derived EVs significantly upregulated ITGB2 in RAW264.7 cells (1.5-fold, *P* < 0.001) (Figure 4, C and D), suggesting orchestrated transcriptional regulation of cellular processes that favor cell movement and leucocyte adhesion by EC-EVs. Sham EC-EV preparations had no significant effect on gene expression in mouse (*gapdh*, *cyclophilin*, *actin*, *b2m*, *itgb2*, *ccr2*, and *il-6*) or human monocytes (*GAPDH*, *CYCLOPHILIN*, *ACTIN*, *B2M*, *ITGB2*, *CCR7*, and *IL-6*) (Supplemental Figure 3, A and C). ITGA4 and ITGB2 form subunits of the VLA-4 integrin, the receptor for VCAM-1, which is involved in monocyte-EC recognition and binding (36, 37).

### EC-EV and monocyte interactions through VCAM-1 enhance cell migration to MCP-1.

**Figure 5:**
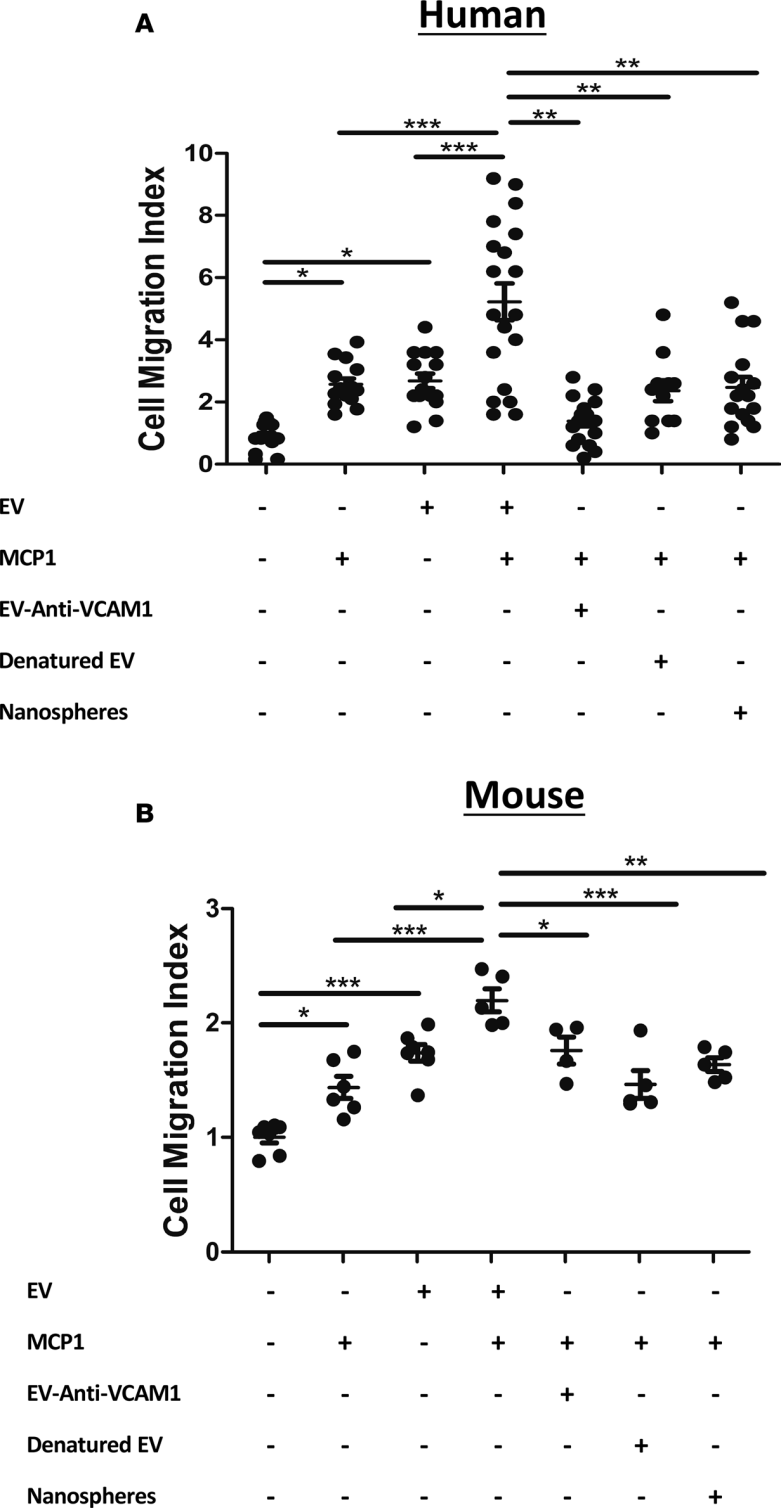
EC-EVs enhance monocyte chemotaxis. Inflammatory HUVEC and sEND.1 EVs enhance (**A**) THP-1 monocyte and (**B**) RAW264.7 monocyte chemotaxis to MCP-1 (50 nM), respectively, an interaction mediated through VCAM-1 (*n* = 4–19 per group). Grouped values are mean ± SEM. One-way ANOVA with post-hoc Bonferroni correction. *P < 0.05, **P < 0.01, ***P < 0.001.

These changes in gene expression implied a possible role for EVs in promoting monocyte mobilization. We therefore tested the effects of EC-derived EVs in promoting monocyte motility/mobilization in vitro. EC-EVs (HUVEC and s.END1, respectively) stimulated THP-1 and RAW264.7 cell chemokinesis (Figure 5) (*P* < 0.01). Furthermore, THP-1 migration increased in response to inflammatory HUVEC-derived EVs (2.5-fold, *P* < 0.001) (Figure 5A). Similarly, RAW264.7 cells displayed significantly increased chemotaxis to MCP-1 when exposed to inflammatory EC-EVs (1.3-fold, *P* < 0.01) (Figure 5A); whereas, Sham EC-EVs had no significant effect on THP-1 or RAW264.7 cell motility (Supplemental Figure 3, B and D). The enhanced migration of THP-1 and mouse RAW 264.7 cells in response to MCP-1 through EC-EV exposure was abolished by preincubating inflammatory EC-EVs with an anti-VCAM-1 antibody (THP-1, *P* < 0.001; RAW 264.7, *P* < 0.05), boiling EC-EVs (THP-1, *P* < 0.001; RAW 264.7, *P* < 0.01), or using inert 100-nm silica spheres instead of EC-EVs (THP-1, *P* < 0.001; RAW 264.7, *P* < 0.01) (Figure 5). Collectively these findings show that VCAM-1–mediated interactions between EC-EV monocytes lead to changes in cellular motility.

### EC-EVs mobilize splenic monocytes in vivo.

**Figure 6:**
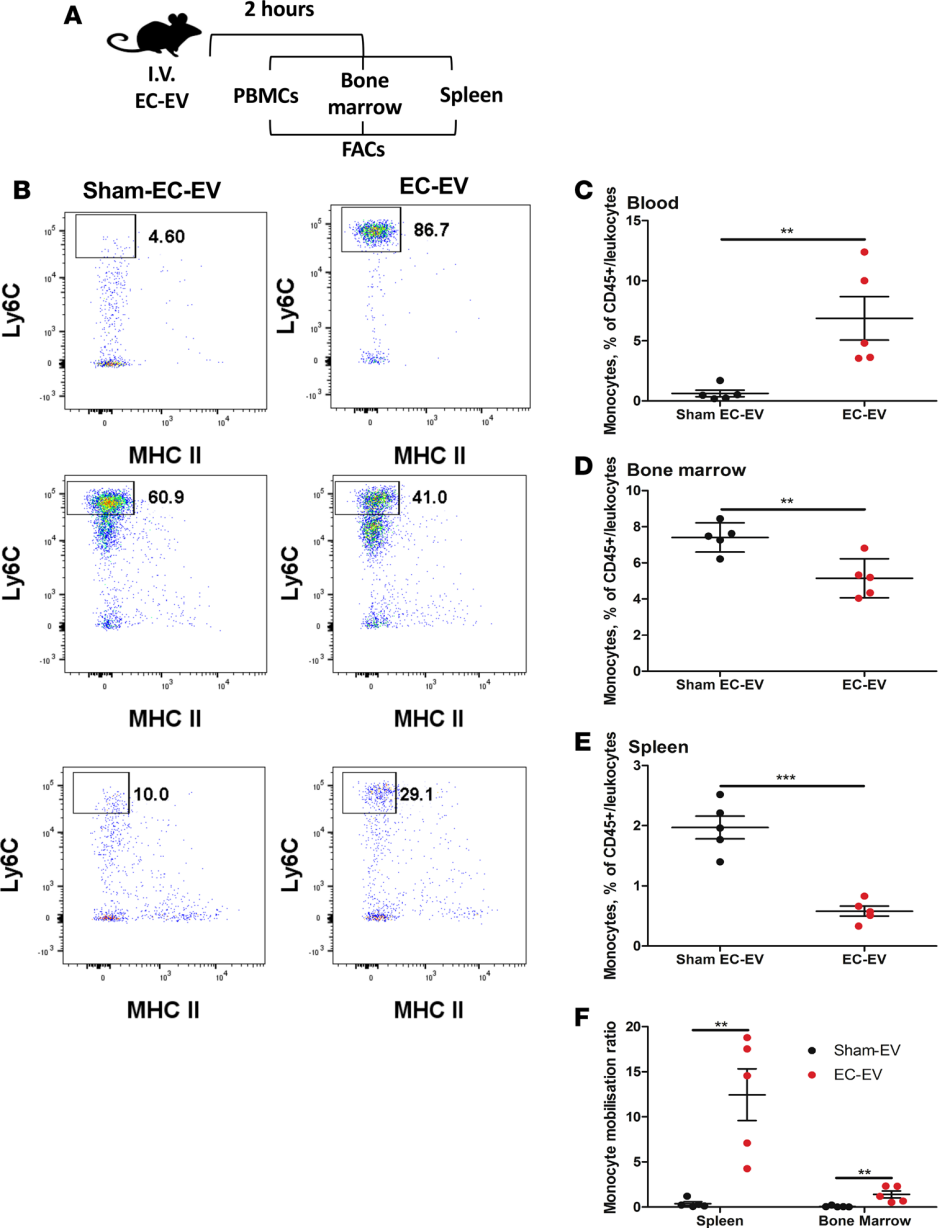
EC-EVs mobilize splenic monocytes. (**A**) EC-EVs were tail vein injected into wild-type mice, and monocyte number was quantified by (**B**) FACS analysis in (**C**) peripheral blood, (**D**) bone marrow, and (**E**) spleen. (**F**) Monocyte mobilization ratio in mice treated with EC-EVs. Values are the percentage of CD45^+^ monocytes (mean ± SEM). Unpaired *t* test (*n* = 5 per group). **P < 0.01, ***P < 0.001.

To better understand the possible physiological relevance of the effects of EC-EVs on monocyte motility, we tested splenic monocyte motility in vivo (Figure 6A). EC-EVs (s.END1) were injected via the tail vein into wild-type mice, and peripheral blood, bone marrow, and spleen were harvested. Following isolation of peripheral blood mononuclear cells (PBMCs), bone marrow cells and splenic digestion, monocyte numbers were quantified by antibody flow cytometry (Figure 6B). EC-EV injection significantly increased monocyte number in peripheral blood (11.1-fold, *P* < 0.01) (Figure 6C) and reduced splenic monocyte number (Figure 6E) in otherwise, healthy naive mice. We found that there is a contribution of bone marrow monocytes to the peripheral monocytosis (Figure 6E) that occurs after i.v. injection of EC-EVs. (1.44-fold, *P* < 0.01). We further calculated a splenic monocyte mobilization ratio (percentage of peripheral blood monocytes/percentage of splenic monocytes or percentage bone marrow monocytes) for each mouse. The ratio quantifies the monocyte mobilization response from different niches in the same mouse. The bone marrow monocyte mobilization by EC-EVs is much smaller in magnitude when compared with the monocyte mobilization that occurs from the spleen in response to EC-EVs (Figure 6F).

## Discussion

Activation and mobilization of monocytes occurs after AMI in mice and humans (1, 2). In mice, blood monocytes peak 24 hours after AMI and are important mediators of tissue injury and repair. Local splenic monocyte mobilization signals may include angiotensin II (2, 7) and β-adrenergic signals stimulation (9). It is not known how ischemic myocardium signals to trigger both mobilization and activation of monocytes, yet regulation of monocyte mobilization, activation, and function at their site of infiltration presents important therapeutic opportunities after AMI.

The principal findings of this study were that (a) total EV number increases after AMI in mice and in humans, where the increase was proportional to the extent of ischemic injury; (b) the increase in plasma EVs is largely accounted for by EC-EVs that bear VCAM-1 and which are enriched for several miRNA, including the EC-associated miR-126-3p and miR-126-5p; and (c) injected EVs localize to the spleen and mobilize splenic monocytes.

EVs of mixed cellular origins are present in the plasma of healthy individuals, and circulating levels are altered in many pathophysiological conditions (38). Ischemic syndromes are characterized by arterial occlusion, but venous endothelium still has access to the blood and is a potential source of EV. By isolating the VCAM-1–positive EVs using antibody-coated magnetic particles, we showed that VCAM-1–bearing EVs increased approximately 3-fold after AMI. Contributions from other sources, e.g., activated platelets cannot be excluded by this approach, but the presence of VCAM-1 in plasma-derived EVs supports the conclusion that ECs contribute substantially. Furthermore, by contrast to VCAM-1, we did not identify platelets or platelet-derived EVs using anti-CD41 antibodies in vivo.

VCAM-1 is fundamental to the recruitment of blood monocytes to sites of endothelial activation and contributes to leukocyte recruitment in atherosclerosis and AMI (39–41). Human coronary ECs express greater VCAM-1 in AMI and release microparticles, which positively correlate with vascular inflammation (42). Here, we show that ECs release EVs that themselves bear VCAM-1 under basal and inflammatory conditions, signaling monocyte movement. EC integrins are important in monocyte adhesion and motility.

Various cell types, including monocytes and macrophages, avidly internalize plasma EVs (30, 43), and EV-associated integrins have demonstrated the ability to direct EVs to specific organs (44). In agreement with previous observations (26, 30, 45), we found that injected EVs were rapidly cleared from the systemic circulation by the spleen. Here, we show that the splenic EV clearance is not merely a disposal function, but that EVs signal to monocytes, resulting in their mobilization into the blood.

We have previously shown patterns of transcriptional activation of monocytes in the peripheral blood following AMI that are conserved between mice and humans (1) indicative of transcriptional programming prior to entry into the infarcted myocardium. We reasoned that the RNA contained within EV could contribute to this transcriptional programming. Compared with changes in whole plasma, EVs are enriched for a smaller number of miRNAs, and EV-associated protein (tetraspanins, TSG101, and ALIX) appears to be conserved across various cell types (46); however, protein and nucleic acid profiles of EC-EVs change considerably to reflect differential stimuli (47, 48). miRNA have shown regulation of inflammatory responses in monocytes and macrophages (49–52). Here, we have shown that a relatively small number of miRNAs are enriched in plasma EVs and are altered significantly after AMI. miRNA-126-3p and miRNA-126-5p were highly represented and changed after AMI in our investigations. EC-associated precursor miRNA-126 gives rise to the mature miR-126-5p and miR-126-3p, which are among the most abundant miRNA in ECs (53, 54), adding weight to the interpretation that the surge in EVs in AMI is derived from ECs. Furthermore, consistent with current findings, miR-126 has previously been shown to have roles in the mobilization of hematopoietic progenitor cells by reducing the expression of VCAM-1 (55). After myocardial infarction, miRNA-126 may have additional roles in the regulation of vascular integrity and neovascularization (31). Genetic depletion of miRNA-126-5p promotes EC proliferation and limits atherosclerosis in apolipoprotein E–deficient mice (54).

miRNA-mRNA pathway analysis identified cellular growth and proliferation targets. Prior investigations in our laboratory have identified proliferation pathways as important regulators of monocyte function after AMI (1), and this may be mediated by EV-miRNAs, as identified in this study. EV miRNAs may mediate monocyte production after AMI locally in the spleen or in the bone marrow. However, available data on the majority of these miRNAs are in cell types (56) that are not relevant to our investigations. Identification of miRNA that regulates the inflammatory phase of monocyte cell recruitment and activation after injury may allow novel strategies for both diagnosis and therapy to be explored. Given their relationship to infarct size and monocyte mobilization, it is conceivable that analysis of VCAM-1–positive EVs could be a useful tool for stratification and selection of rational therapies in human AMI. Current treatments for AMI focus disproportionately on reperfusion therapies, which (to be effective) must be delivered within the first hours of AMI. Conversely, it seems that for a given ischemic insult, immune cell infiltration propagates later injury (1, 5, 7, 57) and represents a target for therapy in its own right (6, 9). Engineered EVs bearing selective miRNA may allow monocyte transcriptomes to be modulated by inhibiting the proinflammatory phase of monocyte recruitment and activation (which is otherwise associated with tissue injury) and synergistically promoting pathways for repair and regeneration. This could potentially extend the time window for effective treatments in humans up to 48 hours after AMI, in which peripheral monocytosis arises (1).

Therapeutic targeting of EV-associated VCAM-1 with anti-VCAM-1 antibodies after AMI may limit splenic monocyte cell movement. The creation and use of bioengineered EVs that are systematically administered into the plasma at the time of AMI may allow therapeutic manipulation of the EV–VCAM-1 axis for specific targeting of monocytes.

### Limitations.

Patients experiencing AMI in our investigations underwent primary percutaneous coronary intervention, in which coronary blood flow is restored following initial coronary artery occlusion and myocardial ischemic injury. Reperfusion therapies in AMI are associated with the onset of inflammation and EC activation (58), which could further stimulate EC-EV release. In contrast, our mouse model of AMI used permanent ligation of the coronary microvasculature without reperfusion. This permanent ligation method of experimental AMI may evoke greater inflammatory-mediated injury in the mouse (59). Importantly, the contribution made by ECs to the flux in plasma EV number after AMI may be highly regulated by the onset of inflammation in the myocardium, such as the local and sustained released of TNF-α. Uncovering differences in plasma EV profiles between the ligation and reperfusion models of rodent AMI may enhance our understanding of how monocytes are mobilized and recruited from the spleen and may uncover the disease model discrepancies in rodent myocardial pathophysiology (59).

### Conclusions.

Taken together, the findings in this study demonstrate that plasma-liberated EVs signal the mobilization and transcriptional activation of monocytes after AMI. Plasma EV levels significantly increase after AMI and correlate closely with the degree of injury in humans. ECs release EVs, and inflammatory stimulation significantly increases EV release. EC-EVs from stimulated cells display a EV–VCAM-1 enrichment, localize to the spleen, and interact with and mobilize splenic monocytes. EC-EV exposure significantly alters monocyte motility–associated genes and shows regulation of integrins that favor tissue recruitment. Our study has unveiled a mechanism by which the ischemic myocardium signals both monocyte mobilization and transcriptional activation after AMI. This study provides a paradigm for inhibiting monocyte mobilization and activation after injury and favorable modulation of the inflammatory phase of heart injury, which is otherwise associated with attenuated cardiac function and poor long-term prognosis.

## Methods

### Human plasma isolation and MRI scanning.

Plasma was obtained from 22 patients presenting at the Oxford Heart Centre with ST-segment elevation AMI, as part of the OxAMI Study (REC number 10/H0408/24) (1). Peripheral venous blood was obtained from patients at presentation in k2-ethylenediaminetetraacetic acid–coated (K2-EDTA–coated) tubes (BD Vacutainer) and processed within 30 minutes. Platelet-poor plasma was obtained by two rounds of centrifugation (5,000 *g* for 10 minutes). Plasma samples were frozen and stored at −80°C. All patients (*n* = 22) underwent 3 Tesla cardiac magnetic resonance imaging (Verio, Siemens, Germany) within 48 hours of presentation (1). Cardiac magnetic resonance analysis was carried out using cmr42 software (Circle Cardiovascular Imaging Inc.).

### Mouse myocardial infarction and plasma EV.

Wild-type adult C57BL/6 mice (Harlan) were used in all investigations (12–18 weeks of age). Experimental AMI was performed in female mice due to an increased mortality rate in male mice. Surgical AMI was induced by permanent ligation of the coronary artery as previously described (1). Researchers were blinded to group allocations for the duration of the experiment. Whole blood samples were collected by cardiac puncture under terminal anesthesia and centrifuged in spray-coated K2-EDTA blood collection tubes (BD Vacutainer). Platelet-poor plasma was obtained by two rounds of centrifugation (5,000 *g* for 10 minutes). Plasma samples were frozen and stored at −80°C.

### EV isolation.

EVs were isolated using differential centrifugation (60). Briefly, cell culture supernatants and platelet-poor plasma were centrifuged at 300 *g* for 10 minutes. The resultant supernatant was transferred to ultracentrifuge tubes (Polyallomer Quick-Seal ultra-clear 16 mm × 76 mm tubes, Beckman Coulter) and centrifuged at 120,000 *g* for 120 minutes to pellet all EVs. Isolated vesicles were resuspended in 100 μl PBS (ThermoFisher Scientific) and utilized in studies as described. EVs were characterized by nanoparticle tracking analysis (NTA) (61), TEM, and protein was characterized by Western blotting.

### EV characterization.

EV size and concentration profiles were determined by using a Nanosight NS500 instrument (Malvern), sCOMS camera, and NTA software version 2.3, build 0033. Silica 100-nm microspheres (Polysciences Inc.) were routinely analyzed to quality check instrument performance. EVs were prepared for NTA analysis by dilution in PBS at approximately 3 × 10^8^ EV/ml to 5 × 10^8^ EV/ml. EV preparations were loaded into the sample chamber and analyzed according to the following measurement script: prime; 5-second delay; 30-second capture; measurements were repeated 5 times per sample. The camera gain was set to level 12 for all measurements (camera shutter speed, 15 milliseconds; camera gain, 350 milliseconds). Acquisitions were analyzed using the proprietary software.

### TEM.

TEM of isolated EVs was conducted as previously described (62). Briefly, 5–10 μl of 0.1 mg/ml EVs was fixed with 4% paraformaldehyde (PFA) and allowed to settle (1 minute) onto pyroxylin- and carbon-coated copper grids (400 mesh, Agar Scientific). Grids were then blotted dry with filter paper and washed with Milli–Q water. For negative staining, 2% (w/v) uranyl acetate (10 seconds) was used, the excess solution was removed with filter paper, and the grids were left to air dry before imaging. Grids were viewed at 120 kV in an FEI Tecnai12 TEM (FEI UK Ltd), and images were obtained using a bottom-mounted Gatan OneView camera.

### Western blot of EV.

Western blotting for the EV markers CD63, CD9, and HSP70 (EXOAB-KIT-1, System Biosciences), TSG101 (ab83, Abcam), and ALIX (ab117600, Abcam) was undertaken as previously described by Lötvall et al. (63). The purity of EV samples was tested for contaminating intracellular organelles by ATP5A (mitochondria) (15H4C4, Abcam), nuclear fractions by histones (histone H3) (D1H2/4499P, Cell signaling), and platelets by CD41 (ab63983, Abcam). Briefly, protein quantity in EVs was detected by lysing EV pellets in CelLytic MT Cell LysisReagent or RIPPA buffer (Cell Signaling) with added protease inhibitors (Roche Complete). To ensure equal loading, protein concentration was measured by bicinchoninic acid assay (Thermo Scientific), and 10–40 μg protein was loaded per well. SDS page samples were separated on 4%–12% bis-tris gradient gels (Life Technologies) under nonreducing conditions. Separated samples were transferred to PVDF or nitrocellulose membranes, and nonspecific binding was blocked with 5% milk in PBS-Tween. Membranes were incubated with antibodies overnight, and membranes were washed and incubated with a secondary antibody conjugated to horseradish peroxidase. Membranes were washed again before incubation with enhanced chemiluminescence substrate (Pierce ECL, ThermoFisher Scientific) and imaged on a Bio-Rad ChemiDoc MP Imaging system.

### Cell culture and reagents for EC-EV generation and isolation.

EV experiments were performed in EV-depleted media prepared by ultracentrifugation for respective cell culture mediums (below) at 120,000 *g* for 18 hours (Beckman Coulter, Optima MAX-XP Ultracentrifuge). All cell cultures were maintained in a humidified atmosphere of 95% air/5% CO_2_ at 37°C and stimulated for the generation of EVs when at 80% confluence in 13 ml medium in T75 tissue culture flasks (Sigma-Aldrich).

HUVECs (Lonza) were cultured in EGM-2 supplemented with BulletKit and 2% FBS (Lonza). Cells were treated with cytokines for 16 hours (10 ng/ml TNF-α; 10 ng/ml IL-1β; 1 ng/ml IL-4 or 10 ng/ml IL-6; R&D Systems) for EV generation. Sham EVs were prepared by culturing EV-depleted medium without cells for 16 hours. THP-1 human monocytes (ATCC) were cultured in RPMI medium (Sigma-Aldrich) supplemented 10% FBS, 1% antibiotics (Penicillin-Streptomycin) (Sigma Aldrich), 2 mM L-glutamine (Sigma-Aldrich), and 0.05 nM 2-mercaptoethanol. Monocytes were differentiated into macrophages by exposure to phorbol myristate acetate (PMA) (200 nM) for 24 hours. Similarly, RAW264.7 (ATCC) cells were cultured in RPMI-1640 supplemented with 10% FBS, 1% antibiotics (Penicillin-Streptomycin), and 2 mM L-glutamine. Primary cardiac mouse microvascular ECs (Cell Biologics) and the murine endothelioma sEND.1 (gift from K.M. Channon, University of Oxford) (64) cell line were cultured in speciality complete EC medium with supplements (Cell Biologics) or Dulbecco’s modified eagle’s medium (DMEM) (Sigma-Aldrich) supplemented with 10% FBS, 1% antibiotics (Penicillin-Streptomycin), and 2 mM L-glutamine, respectively. Mouse ECs were treated with 10 ng/ml recombinant mouse TNF-α, 10 ng/ml recombinant mouse IL-4, or 10 ng/ml recombinant mouse IL-6 (R&D Systems) for 16 hours for EV generation. Primary cardiomyocytes were isolated as previously described (65, 66) from wild-type C57B/6J male mice. Briefly, whole hearts were Liberase digested by retrograde perfusion using a Langendorff system and seeded onto glass coverslips. Cells were rested for 2 hours prior to 10 ng/ml TNF-α stimulation in EV-depleted medium for 16 hours. Cells were seeded into tissue culture wells for described stimulation studies.

### Luminex multiplex assays and ELISA.

Human adhesion molecule Luminex performance assay 4-plex (ICAM-1, E selectin, P selectin, and VCAM-1) (R&D Systems, ), mouse VCAM-1/CD106 (R&D Systems), and Milliplex MAP mouse cardiovascular disease bead panel (sP selectin, PECAM-1, sICAM-1, sE selectin) were used according to the manufacturer’s instructions for the detection of EV EC markers.

### Anti-VCAM-1 magnetic particle quantification of EV.

Goat anti-mouse VCAM-1 (AF643, R&D Systems, AF643) or normal goat IgG control (AB-108-C, R&D Systems) antibodies were covalently conjugated to M-450 Tosylactivated Dynabeads as previously described with abundant PBS washing (67). Plasma EVs were isolated from 100 μl platelet-poor plasma and resuspended in 300 μl PBS. 150 μl of each isolated plasma EV preparation was incubated with either anti-VCAM-1 or IgG control conjugated beads at room temperature for 2 hours with continual rotation in PBS (1 ml total). After incubation, magnetic beads were pelleted for 5 minutes using a Dynal Magnet (Invitrogen), and the supernatant containing unbound EVs was subject to NTA for quantification (number of EVs × 10^8^/ml). The percentage of VCAM-1–positive plasma EVs was determined using the number of EVs remaining after IgG control–magnetic bead incubation and the number of EVs remaining after anti-VCAM-1 incubation, as quantified by NTA.

### mRNA and miRNA.

mRNA was isolated from cells using RNeasy Plus Universal Kits (Qiagen) and reverse transcribed using Qiagen Onestep RT-PCR kits (Qiagen). mRNA levels were determined by quantitative PCR (qPCR) using TaqMan probes and reagents. The cyclophilin gene was used as a normalization control and experimental data were compared with EV-treated samples as described. A pathway focused RT^2^ Profiler PCR Array for Human Cell Motility array (Qiagen) was performed according to the manufacture’s instructions (GEO accession no. GSE101159). miRNAs were prepared using miRCURY Cells and Tissue RNA, Biofluids and Cell and Plant isolation kits, and Universal cDNA was synthesis kits II (Exiqon), and transcript levels were quantified using miRCURY LNA microRNA PCR, ExiLENT SYBR master mix with ready-to-use PCR, human panel I and II or cel-miR-39-3p, LNA control primer set kits (Exiqon) according to the manufacture’s protocol.

### miRNA analysis.

In order to separately measure the effects of time point and other variables on miRNA levels, we used an Elastic Net model with iterative fitting along a regularization path. This approach reduces the risk of overfitting by combining the penalties of the lasso and ridge methods and using cross-validation to choose a limited set of predictive variables (with a maximum of 10,000 iterations). EV density was log_2_ transformed to put it on an approximately comparable scale to Ct values. Missing Ct values were not used in the regressions (no imputation), and 24 of 32 (75%) or more of the samples were required to have a measurement. We used the “linear_model.ElasticNetCV” function from scikit-learn. The explained variable was Ct, and the predictor variables were time point (acute or follow-up), patient ID, log_2_ transformed EV density, sex, age, smoker status (1 = smokers, 0.5 = ex-smokers, 0 = nonsmokers), and grams of infarct size. We report here all miRNAs in which the models included time point and where the absolute value of the difference in Ct was greater than or equal to 0.75, as we found that this set was robust to the choice of which clinical measurements were provided to the algorithm as predictor variables, while there was some fluctuation of inclusion among the miRNAs with smaller effect sizes (miRNAs with larger effect sizes were stably reproducible both in terms of inclusion and effect size, regardless of predictor variables provided).

### Ingenuity pathway analysis and GO ontology analysis.

To better understand the role of identified miRNAs in biological pathways, we undertook Ingenuity Pathway Analysis (IPA; https://www.qiagenbioinformatics.com/). Differentially expressed miRNA IDs and expression data (percentage change from the linear elastic model) were imputed to IPA as previously described (68) for miRNA-mRNA target interactions. miRNA-mRNA target interactions were filtered for experimentally observed data, highly and moderately predicted interactions, and pathways listed in IPA. Identified mRNA targets were subjected to core analysis. Furthermore, miRNA-mRNA targets identified by IPA were imputed to GO (http://geneontology.org/) to generate GO terms for biological processes and ranked by fold enrichment.

### Fluorescent immunocytochemistry.

For EV uptake studies, cells (THP-1 PMA-treated monocytes or RAW 264.7) were incubated in exosome-depleted RPMI-1640 supplemented with 2% FBS for a further 24 hours. EVs generated from EC cultures were labeled with PKH67 (Sigma-Aldrich), and macrophages were treated with PKH67-labeled EVs (Green) for specified times. Cell culture media were removed at each time point, and cells were washed in sterile PBS and then fixed using 4% PFA. F-actin was visualized using TRITC-Phalloidin (RED) (Invitrogen). Cells were mounted in glycerol mounting medium with DAPI and DABCO (Blue) (Abcam). Images were acquired using a Zeiss LSM 710 laser-scanning confocal microscope.

### Chemotaxis studies.

Chemotaxis studies were performed using a modified Boyden chamber assay using Neuro Probe ChemoTx plates (Receptor Technologies) with an 8-μm pore size and 20,000–40,000 cells per well, as previously described (69). THP-1 and RAW 264.7 cells were placed above the membrane with and without EC-EVs. The lower well contained media with or without 50 nM recombinant MCP-1 (R&D Systems). The plates were incubated for 90 minutes in a humidified atmosphere of 95% air/5% CO_2_ at 37°C. Following incubation, remaining cells were removed by washing, and the membrane was fixed in 4% PFA for 10 minutes. The membrane was mounted onto slides using DAPI-staining mounting medium (ThermoFisher Scientific). Cell migration was quantified by taking 4 images under a fluorescent microscope (×20 objective) from each well, after which an average was obtained. Stained nuclei were counted using ImageJ software (NIH). Data were normalized to the migration of cells to control media and expressed as fold change (cell migration index).

### Biodistribution of EV.

s.END1 EC-EVs were labeled with *C*. *elegans cel*-miR-39-3p transcript (Exiqon) using an exosomal transfection kit (Exo-Fect, System Bioscience) according to the manufacture’s instructions. EC-EVs were then washed by ultracentrifuging at 120,000 *g* for 120 minutes in 13 ml PBS. Labeled EC-EVs were injected into mice by tail vein injection at a concentration of 1 × 10^9^ EVs, and tissues of interest harvested. Tissues were perfused with heparinized PBS (10 ml) through insertion of a 25-gauge needle into the left ventricle. Tissues and plasma were harvested and snap frozen for qPCR analysis; harvested tissues were mechanically homogenized. To determine the uptake of labeled EVs by resident splenic cell populations (splenic monocytes vs. splenic ECs) isolated spleens were dissociated and splenocytes were incubated with anti-CD31 –conjugated (clone MEC 13.3, BD Pharmingen) magnetic beads (sheep anti-rat IgG Dynabeads, Invitrogen) to isolate splenic ECs as previously described (70). The resultant supernatant containing unbound cells was enriched for monocyte populations using EasySep (Stemcell Technologies) as previously described (1). Isolated cells were lysed and prepared for miRNA RT-qPCR.

### Monocyte FACS analysis.

EC-EVs were injected into mice by tail vein injection at a concentration of 1 × 10^9^ EVs. Blood was collected by cardiac puncture in K2-EDTA–coated tubes 2 hours after injection and lysed using a red blood cell (RBC) lysis solution at room temperature for 10 minutes. Similarly, spleen was disassociated with a sterile cell strainer (70 μm) and RBCs were lysed. Bone marrow cells were flushed from the femur and tibia with PBS. Cells (PBMCs, bone marrow, and splenocytes) were isolated by centrifugation and resuspended in a FACS buffer (PBS plus 2% FBS).

5 × 10^5^ to 2 × 10^6^ cells were plated on either V-bottomed or U-bottomed 96-well plates. The cells were washed twice with 150 μl FACS buffer (PBS plus 0.1% BSA plus 1 mM EDTA plus 0.01% sodium azide) at 400 *g* for 3 minutes 4°C. Cells were then Fc blocked for 10 minutes with αCD16/CD32 (BD) 1:100 in 20 μl FACS buffer at room temperature followed by washing once in 150 μl FACS buffer. Fixable Viability Dye eFluor 780 (ThermoFisher) and primary extracellular antibodies (Supplemental Table 1) were added at indicated concentrations for 20 minutes at 4°C in 20 μl FACS buffer in the dark. Labeled cells were then washed twice with 150 μl FACS buffer. Cells were then fixed for 30 minutes in 50 μl Cytofix (BD), washed twice with 150 μl FACS buffer, and resuspended in 200 μl FACS buffer before acquisition.

CompBeads (BD) were used to prepare single-stained controls as per the manufacturer’s instructions to set up fluorophore compensation. Fluorescence minus one controls were used to set gates for cell populations. Labeled cells were acquired using either an LSR II (BD) or Fortessa X20 (BD) flow cytometer using FACSDiva (BD). Data were analyzed using Flowjo (Treestar Inc.) software. After doublet and size exclusion Ly6C^hi^ monocytes were defined as live, CD45^+^, CD11b^+^, Ly6G^–^, Siglec, F^–^, MHC II^–^, Ly6C^+^.

### Statistics.

Data are expressed as group mean ± SEM. Within and between experimental group differences were compared using 2-tailed Student’s *t* tests and 1-way ANOVA with post-hoc Bonferroni correction when significant differences were found. Associations between plasma EVs were tested using Pearson correlation coefficients in the software package GraphPad Prism (version 5). Values were considered significant at *P* < 0.05.

### Study approval.

All clinical investigations were conducted in accordance with the Declaration of Helsinki. The Oxfordshire Research Ethics Committee (references 08/H0603/41 and 11/SC/0397) approved human clinical cohort protocols. All patients provided informed written consent for inclusion in the study. All animal procedures were approved by an ethical review committee at the University of Oxford, and experimental interventions were carried out by UK Home Office personal licence holders under the authority of a Home Office project licence.

## Author contributions

NA isolated, characterized, and utilized all mouse-derived EVs in described experiments. JED isolated, characterized, and utilized all human-derived EVs in described experiments. TJC and ALC prepared and analyzed FACS preparations. ANT assisted with cell migration experiments and EV isolations. SS assisted with Western blot preparations. NR isolated primary cardiomyocytes. EJ and AAA prepared and imaged EVs by TEM. RAD assisted with NTA. KMC and SD assisted with miRNA analysis. PRR and DCA led animal investigations. NA, JED, RPC, DCA, KMC, IAU, and PRR conceived the study; participated in its design and coordination; and helped to draft the manuscript. LE, EM, MR, and DRG assisted with experimentation. KZ assisted with image analysis for chemotaxis studies All authors read the final version of the manuscript and approved its submission.

## Supplementary Material

Supplemental data

## Figures and Tables

**Table 3:**
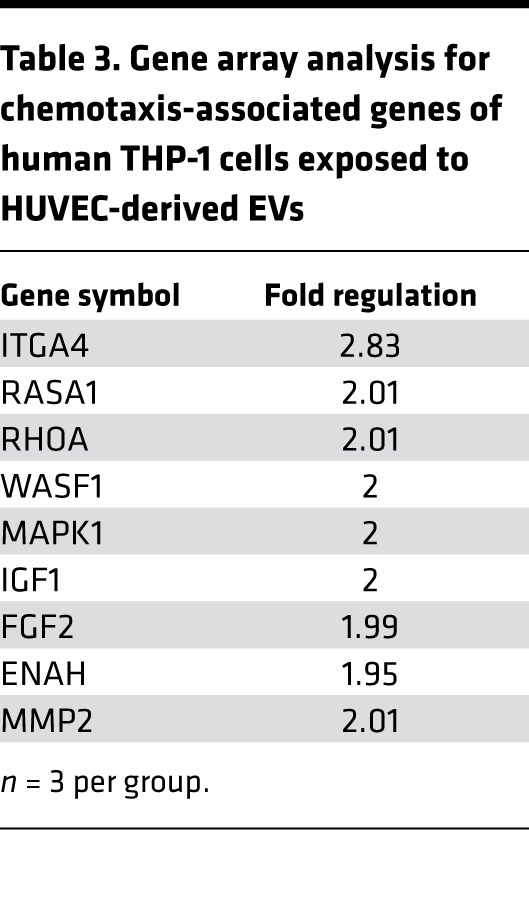
Gene array analysis for chemotaxis-associated genes of human THP-1 cells exposed to HUVEC-derived EVs

**Table 2:**
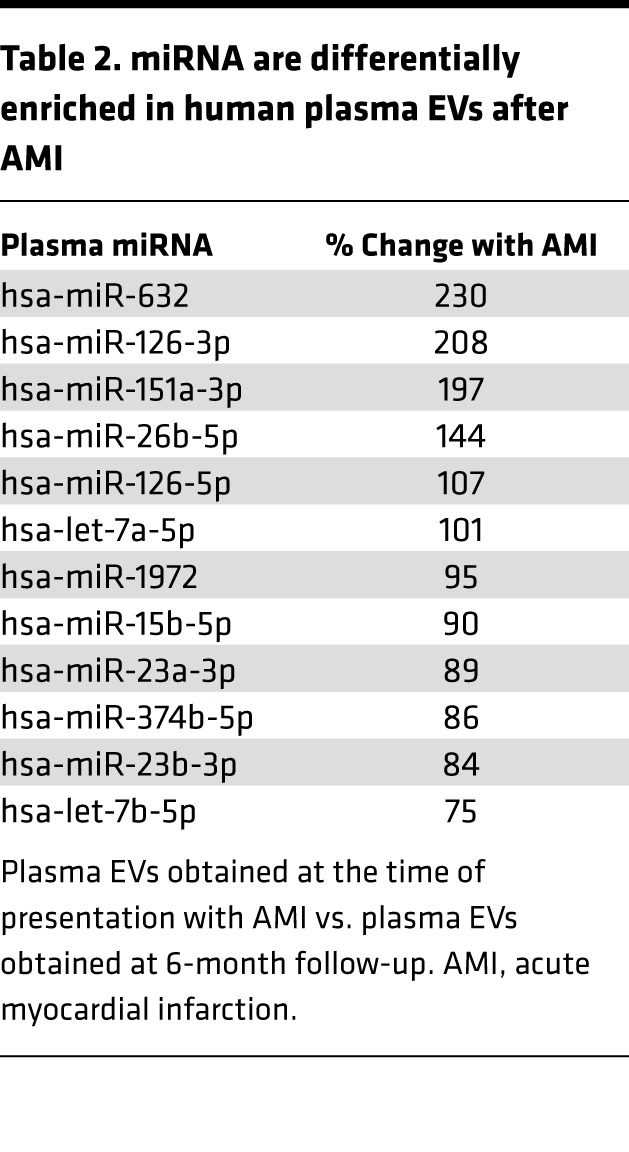
miRNA are differentially enriched in human plasma EVs after AMI

**Table 1:**
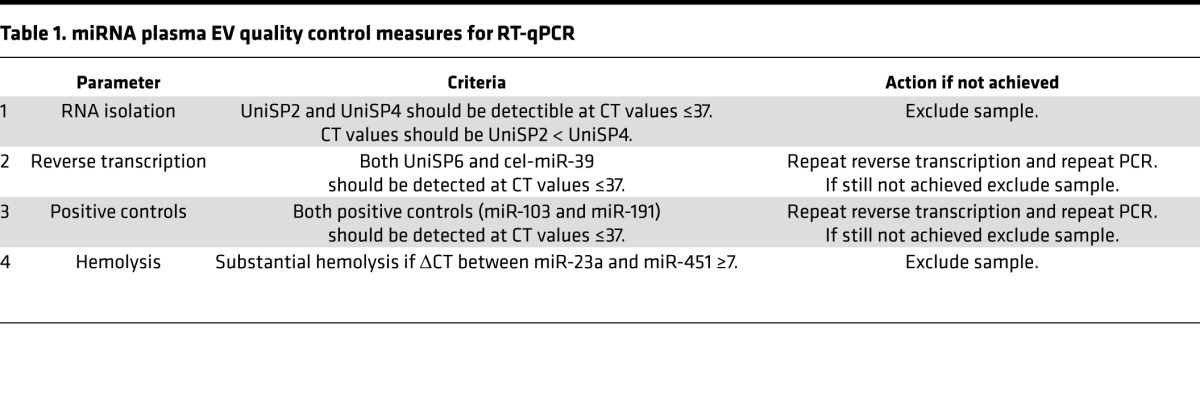
miRNA plasma EV quality control measures for RT-qPCR

## Version 1 08/29/2017

Electronic publication.

## References

[1] Ruparelia N (2015). Acute myocardial infarction activates distinct inflammation and proliferation pathways in circulating monocytes, prior to recruitment, and identified through conserved transcriptional responses in mice and humans. Eur Heart J.

[2] Swirski FK (2009). Identification of splenic reservoir monocytes and their deployment to inflammatory sites. Science.

[3] Panizzi P (2010). Impaired infarct healing in atherosclerotic mice with Ly-6C(hi) monocytosis. J Am Coll Cardiol.

[4] Nahrendorf M (2007). The healing myocardium sequentially mobilizes two monocyte subsets with divergent and complementary functions. J Exp Med.

[5] Leuschner F (2011). Therapeutic siRNA silencing in inflammatory monocytes in mice. Nat Biotechnol.

[6] Majmudar MD (2013). Monocyte-directed RNAi targeting CCR2 improves infarct healing in atherosclerosis-prone mice. Circulation.

[7] Leuschner F (2010). Angiotensin-converting enzyme inhibition prevents the release of monocytes from their splenic reservoir in mice with myocardial infarction. Circ Res.

[8] Leuschner F (2010). Angiotensin-converting enzyme inhibition prevents the release of monocytes from their splenic reservoir in mice with myocardial infarction. Circ Res.

[9] Grisanti LA (2016). Leukocyte-expressed β2-adrenergic receptors are essential for survival after acute myocardial injury. Circulation.

[10] Potteaux S, Tedgui A (2015). Monocytes, macrophages and other inflammatory mediators of abdominal aortic aneurysm. Curr Pharm Des.

[11] Pironti G (2015). Circulating exosomes induced by cardiac pressure overload contain functional angiotensin II type 1 receptors. Circulation.

[12] Salido-Guadarrama I, Romero-Cordoba S, Peralta-Zaragoza O, Hidalgo-Miranda A, Rodríguez-Dorantes M (2014). MicroRNAs transported by exosomes in body fluids as mediators of intercellular communication in cancer. Onco Targets Ther.

[13] Sluijter JP, Verhage V, Deddens JC, van den Akker F, Doevendans PA (2014). Microvesicles and exosomes for intracardiac communication. Cardiovasc Res.

[14] Kowal J, Tkach M, Théry C (2014). Biogenesis and secretion of exosomes. Curr Opin Cell Biol.

[15] Sandvig K, Llorente A (2012). Proteomic analysis of microvesicles released by the human prostate cancer cell line PC-3. Mol Cell Proteomics.

[16] Mittelbrunn M (2011). Unidirectional transfer of microRNA-loaded exosomes from T cells to antigen-presenting cells. Nat Commun.

[17] Villarroya-Beltri C, Gutiérrez-Vázquez C, Sánchez-Madrid F, Mittelbrunn M (2013). Analysis of microRNA and protein transfer by exosomes during an immune synapse. Methods Mol Biol.

[18] Valadi H, Ekström K, Bossios A, Sjöstrand M, Lee JJ, Lötvall JO (2007). Exosome-mediated transfer of mRNAs and microRNAs is a novel mechanism of genetic exchange between cells. Nat Cell Biol.

[19] Cui Y (2011). Overexpression of sterol carrier protein 2 in patients with hereditary cholesterol gallstones. BMC Gastroenterol.

[20] Deregibus MC (2007). Endothelial progenitor cell derived microvesicles activate an angiogenic program in endothelial cells by a horizontal transfer of mRNA. Blood.

[21] van Balkom BW, Eisele AS, Pegtel DM, Bervoets S, Verhaar MC (2015). Quantitative and qualitative analysis of small RNAs in human endothelial cells and exosomes provides insights into localized RNA processing, degradation and sorting. J Extracell Vesicles.

[22] Chakrabortty SK, Prakash A, Nechooshtan G, Hearn S, Gingeras TR (2015). Extracellular vesicle-mediated transfer of processed and functional RNY5 RNA. RNA.

[23] Halkein J (2013). MicroRNA-146a is a therapeutic target and biomarker for peripartum cardiomyopathy. J Clin Invest.

[24] de Vrij J (2015). Glioblastoma-derived extracellular vesicles modify the phenotype of monocytic cells. Int J Cancer.

[25] Lai CP (2014). Dynamic biodistribution of extracellular vesicles in vivo using a multimodal imaging reporter. ACS Nano.

[26] Imai T (2015). Macrophage-dependent clearance of systemically administered B16BL6-derived exosomes from the blood circulation in mice. J Extracell Vesicles.

[27] Dignat-George F, Boulanger CM (2011). The many faces of endothelial microparticles. Arterioscler Thromb Vasc Biol.

[28] Combes V (1999). In vitro generation of endothelial microparticles and possible prothrombotic activity in patients with lupus anticoagulant. J Clin Invest.

[29] Zhang H (2009). Role of TNF-alpha in vascular dysfunction. Clin Sci.

[30] Saunderson SC, Dunn AC, Crocker PR, McLellan AD (2014). CD169 mediates the capture of exosomes in spleen and lymph node. Blood.

[31] Wang S (2008). The endothelial-specific microRNA miR-126 governs vascular integrity and angiogenesis. Dev Cell.

[32] Harris TA, Yamakuchi M, Ferlito M, Mendell JT, Lowenstein CJ (2008). MicroRNA-126 regulates endothelial expression of vascular cell adhesion molecule 1. Proc Natl Acad Sci USA.

[33] Roney KE (2011). Plexin-B2 negatively regulates macrophage motility, Rac, and Cdc42 activation. PLoS ONE.

[34] Meerschaert J, Furie MB (1995). The adhesion molecules used by monocytes for migration across endothelium include CD11a/CD18, CD11b/CD18, and VLA-4 on monocytes and ICAM-1, VCAM-1, and other ligands on endothelium. J Immunol.

[35] Foster GA (2015). CD11c/CD18 signals very late antigen-4 activation to initiate foamy monocyte recruitment during the onset of hypercholesterolemia. J Immunol.

[36] Tiisala S, Hakkarainen M, Majuri ML, Mattila PS, Mattila P, Renkonen R (1993). Down-regulation of monocytic VLA-4 leads to a decreased adhesion to VCAM-1. FEBS Lett.

[37] Chuluyan HE, Issekutz AC (1993). VLA-4 integrin can mediate CD11/CD18-independent transendothelial migration of human monocytes. J Clin Invest.

[38] Yáñez-Mó M (2015). Biological properties of extracellular vesicles and their physiological functions. J Extracell Vesicles.

[39] Cybulsky MI (2001). A major role for VCAM-1, but not ICAM-1, in early atherosclerosis. J Clin Invest.

[40] Dansky HM (2001). Adhesion of monocytes to arterial endothelium and initiation of atherosclerosis are critically dependent on vascular cell adhesion molecule-1 gene dosage. Arterioscler Thromb Vasc Biol.

[41] Ozaki K (2002). Functional SNPs in the lymphotoxin-alpha gene that are associated with susceptibility to myocardial infarction. Nat Genet.

[42] Radecke CE, Warrick AE, Singh GD, Rogers JH, Simon SI, Armstrong EJ (2015). Coronary artery endothelial cells and microparticles increase expression of VCAM-1 in myocardial infarction. Thromb Haemost.

[43] Feng D (2010). Cellular internalization of exosomes occurs through phagocytosis. Traffic.

[44] Hoshino A (2015). Tumour exosome integrins determine organotropic metastasis. Nature.

[45] Lespagnol A (2008). Exosome secretion, including the DNA damage-induced p53-dependent secretory pathway, is severely compromised in TSAP6/Steap3-null mice. Cell Death Differ.

[46] Théry C (2011). Exosomes: secreted vesicles and intercellular communications. F1000 Biol Rep.

[47] Di Muzio A (2003). Hepatitis C virus infection and myositis: a virus localization study. Neuromuscul Disord.

[48] Song J, Chen X, Wang M, Xing Y, Zheng Z, Hu S (2014). Cardiac endothelial cell-derived exosomes induce specific regulatory B cells. Sci Rep.

[49] Liu G, Abraham E (2013). MicroRNAs in immune response and macrophage polarization. Arterioscler Thromb Vasc Biol.

[50] Urbich C, Kuehbacher A, Dimmeler S (2008). Role of microRNAs in vascular diseases, inflammation, and angiogenesis. Cardiovasc Res.

[51] Yamakuchi M (2012). MicroRNAs in Vascular Biology. Int J Vasc Med.

[52] Zernecke A (2012). MicroRNAs in the regulation of immune cell functions--implications for atherosclerotic vascular disease. Thromb Haemost.

[53] Boon RA, Dimmeler S (2014). MicroRNA-126 in atherosclerosis. Arterioscler Thromb Vasc Biol.

[54] Schober A (2014). MicroRNA-126-5p promotes endothelial proliferation and limits atherosclerosis by suppressing Dlk1. Nat Med.

[55] Salvucci O (2012). MicroRNA126 contributes to granulocyte colony-stimulating factor-induced hematopoietic progenitor cell mobilization by reducing the expression of vascular cell adhesion molecule 1. Haematologica.

[56] Du JY, Wang LF, Wang Q, Yu LD (2015). miR-26b inhibits proliferation, migration, invasion and apoptosis induction via the downregulation of 6-phosphofructo-2-kinase/fructose-2,6-bisphosphatase-3 driven glycolysis in osteosarcoma cells. Oncol Rep.

[57] Kim E, Yang J, Beltran CD, Cho S (2014). Role of spleen-derived monocytes/macrophages in acute ischemic brain injury. J Cereb Blood Flow Metab.

[58] Singhal AK, Symons JD, Boudina S, Jaishy B, Shiu YT (2010). Role of endothelial cells in myocardial ischemia-reperfusion injury. Vasc Dis Prev.

[59] Gao E (2010). A novel and efficient model of coronary artery ligation and myocardial infarction in the mouse. Circ Res.

[60] Cvjetkovic A, Lötvall J, Lässer C (2014). The influence of rotor type and centrifugation time on the yield and purity of extracellular vesicles. J Extracell Vesicles.

[61] Dubuc G (2004). Statins upregulate PCSK9, the gene encoding the proprotein convertase neural apoptosis-regulated convertase-1 implicated in familial hypercholesterolemia. Arterioscler Thromb Vasc Biol.

[62] Théry C, Amigorena S, Raposo G, Clayton A (2006). Isolation and characterization of exosomes from cell culture supernatants and biological fluids. Curr Protoc Cell Biol.

[63] Lötvall J (2014). Minimal experimental requirements for definition of extracellular vesicles and their functions: a position statement from the International Society for Extracellular Vesicles. J Extracell Vesicles.

[64] Williams RL, Risau W, Zerwes HG, Drexler H, Aguzzi A, Wagner EF (1989). Endothelioma cells expressing the polyoma middle T oncogene induce hemangiomas by host cell recruitment. Cell.

[65] Louch WE, Sheehan KA, Wolska BM (2011). Methods in cardiomyocyte isolation, culture, and gene transfer. J Mol Cell Cardiol.

[66] Lomas O, Brescia M, Carnicer R, Monterisi S, Surdo NC, Zaccolo M (2015). Adenoviral transduction of FRET-based biosensors for cAMP in primary adult mouse cardiomyocytes. Methods Mol Biol.

[67] McAteer MA (2007). In vivo magnetic resonance imaging of acute brain inflammation using microparticles of iron oxide. Nat Med.

[68] Shvedova AA, Yanamala N, Kisin ER, Khailullin TO, Birch ME, Fatkhutdinova LM (2016). Integrated analysis of dysregulated ncRNA and mRNA expression profiles in humans exposed to carbon nanotubes. PLoS One.

[69] Gaceb A, Martinez MC, Andriantsitohaina R (2014). Extracellular vesicles: new players in cardiovascular diseases. Int J Biochem Cell Biol.

[70] Sobczak M, Dargatz J, Chrzanowska-Wodnicka M (2010). Isolation and culture of pulmonary endothelial cells from neonatal mice. J Vis Exp.

