# Supplementary material for: Endothelium-derived extracellular vesicles promote splenic monocyte mobilization in myocardial infarction
Source: JCI Insight. 2017 Sep 7;2(17):e93344. doi: 10.1172/jci.insight.93344 (PMC5621885; doi:10.1172/jci.insight.93344)
Supplement: Supplemental data [file jciinsight-2-93344-s001.pdf]

Supplementary Figure 1 (A/B) Nanoparticle Tracking Analysis (NTA) of human plasma extracellular vesicles (EV) at time of presentation with acute myocardial infarction (AMI) and 6 month follow-up (n = 22). (C/D) NTA of primary cardiac mouse myocardial endothelial cell EV released under basal and inflammatory stimulated conditions: TNF-α, IL-4 and IL-6 (n = 3-7). (E/F) NTA of primary mouse cardiomyocytes produced under basal and inflammatory (TNF-α) stimulated conditions (n = 6). Grouped values are means ± SEM. One-way ANOVA with post-hoc Bonferroni correction, paired and unpaired T-test. **P < 0.01, ***P < 0.001.

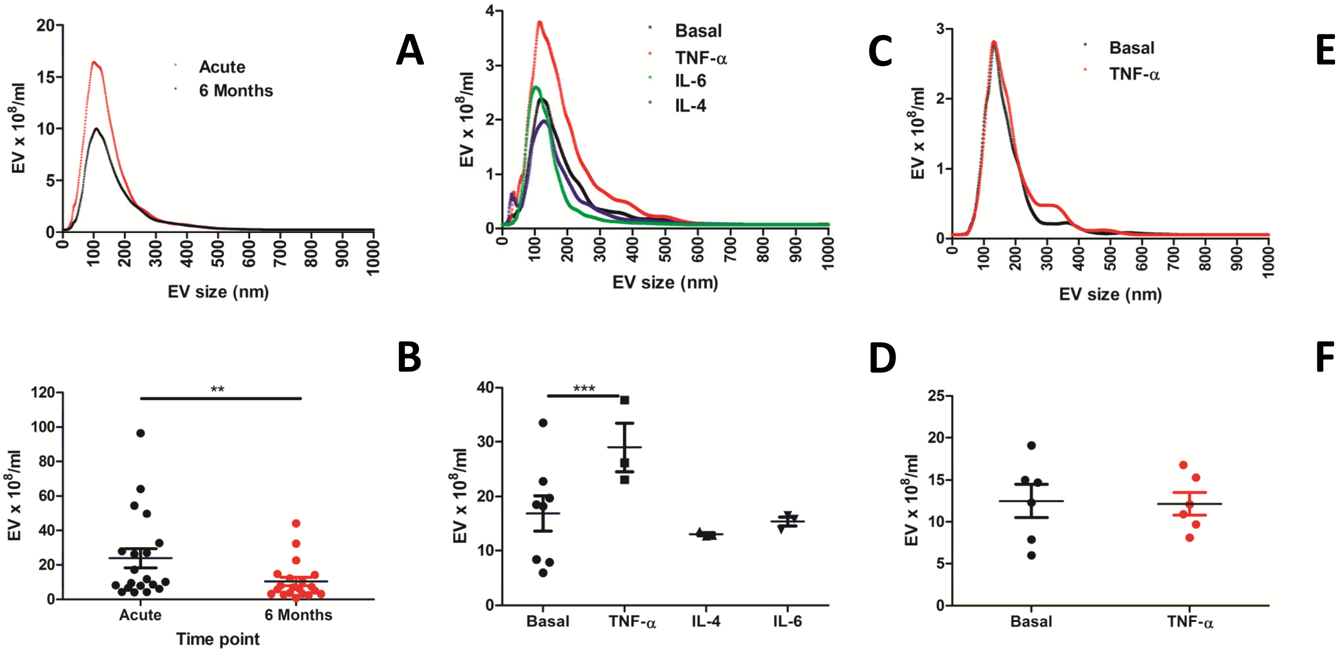

Supplementary Table 1 Antibodies used in FACs analysis of mouse monocytes.

**Antibodies**	**Source**	**Identifier**
Anti-CD45 BV650 (clone 30-F11)	BioLegend	Cat# 103151<br>RRID:AB_2565884
Anti-CD11b BV510 (clone M1/70)	BioLegend	Cat# 101245<br>RRID:AB_2561390
Anti-Ly6G Percp-Cy5.5 (clone 1A8)	BioLegend	Cat# 127615<br>RRID:AB_1877272
Anti-Siglec F BV421 (clone E50-2440)	BD	Cat# 562681
Anti-MHC II (IA/IE) Alexa Fluor 700 (clone M5/114.15.2)	eBioscience	Cat# 56-5321-82<br>RRID:AB_494009
Anti-Ly6C PE-Cy7 (clone HK1.4)	eBioscience	Cat# 25-5932-82
Chemicals, Peptides, and Recombinant Proteins	Source	Identifier
Fixable viability dye eFluor 780	eBioscience	Cat# 65-0865-14

Supplementary Figure 2 (A/B) Nanoparticle Tracking Analysis (NTA) of HUVEC-extracellular vesicles (EV) and HUVEC-sham (n = 4). (C) Protein concentration of HUVEC-EV and HUVEC-sham (n = 4). (D) HUVEC-EV and HUVEC sham Coomassie stained proteins separated on an SDS page gel. (E) Western blot of HUVEC-EV and HUVEC-sham for EV markers, ALIX, TSG101 and CD9. (F/G) NTA of s.END1-EV and s.END1-sham (n = 4). (H) Protein concentration of s.END1-EV and s.END1-sham (n = 8). (I) s.END1-EV and s.END1 sham Coomassie stained proteins separated on an SDS page gel. (J) Western blot of s.END1-EV and s.END1-sham for EV markers, ALIX, TSG101 and CD9. Grouped values are means ± SEM. Unpaired T-test. ***P < 0.001.

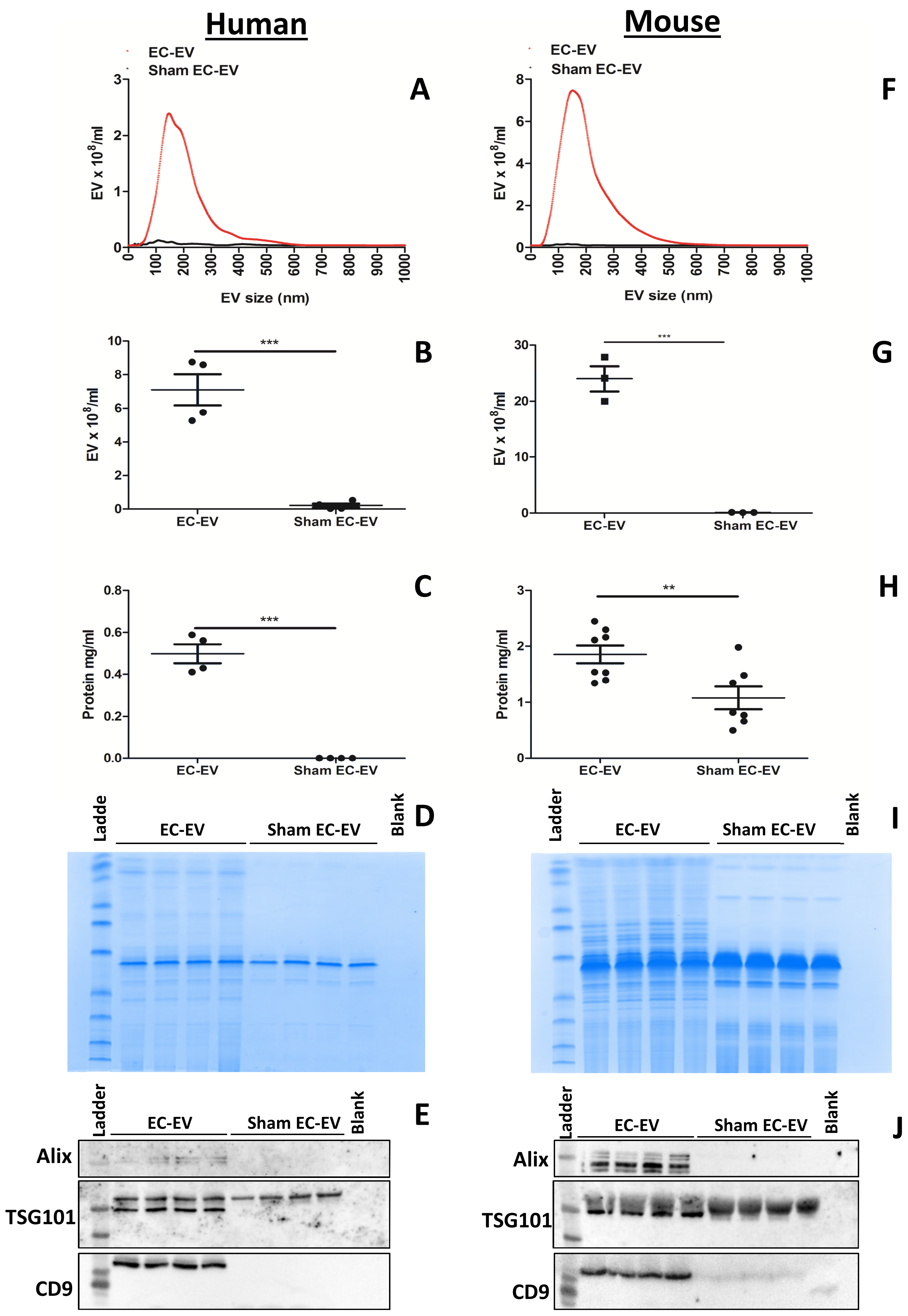
Human
Mouse
EC-EV
Sham EC-EV
A
F
EV x 10^8/ml
EV size (nm)
B
G
***
**
C
H
Protein mg/ml
D
I
Ladder
Blank
E
J
Alix
TSG101
CD9

Supplementary Figure 3

(**A**) Gene expression (Ct values) of THP-1 cells exposed to HUVEC-sham (n=5-8). (**B**) THP-1 cell migration to HUVEC-EV and HUVEC -ham (n = 3-6). (**C**) Gene expression (Ct values) of RAW264.7 cells exposed to s.END1-sham (n=5-8). (**D**) RAW264.7 cell migration to s.END1-EV and s.END1-sham (n = 7-8). Grouped values are means ± SEM. One-way ANOVA with post-hoc Bonferroni correction, paired and unpaired T-test.*P <0.05, **P < 0.01, ***P < 0.001.

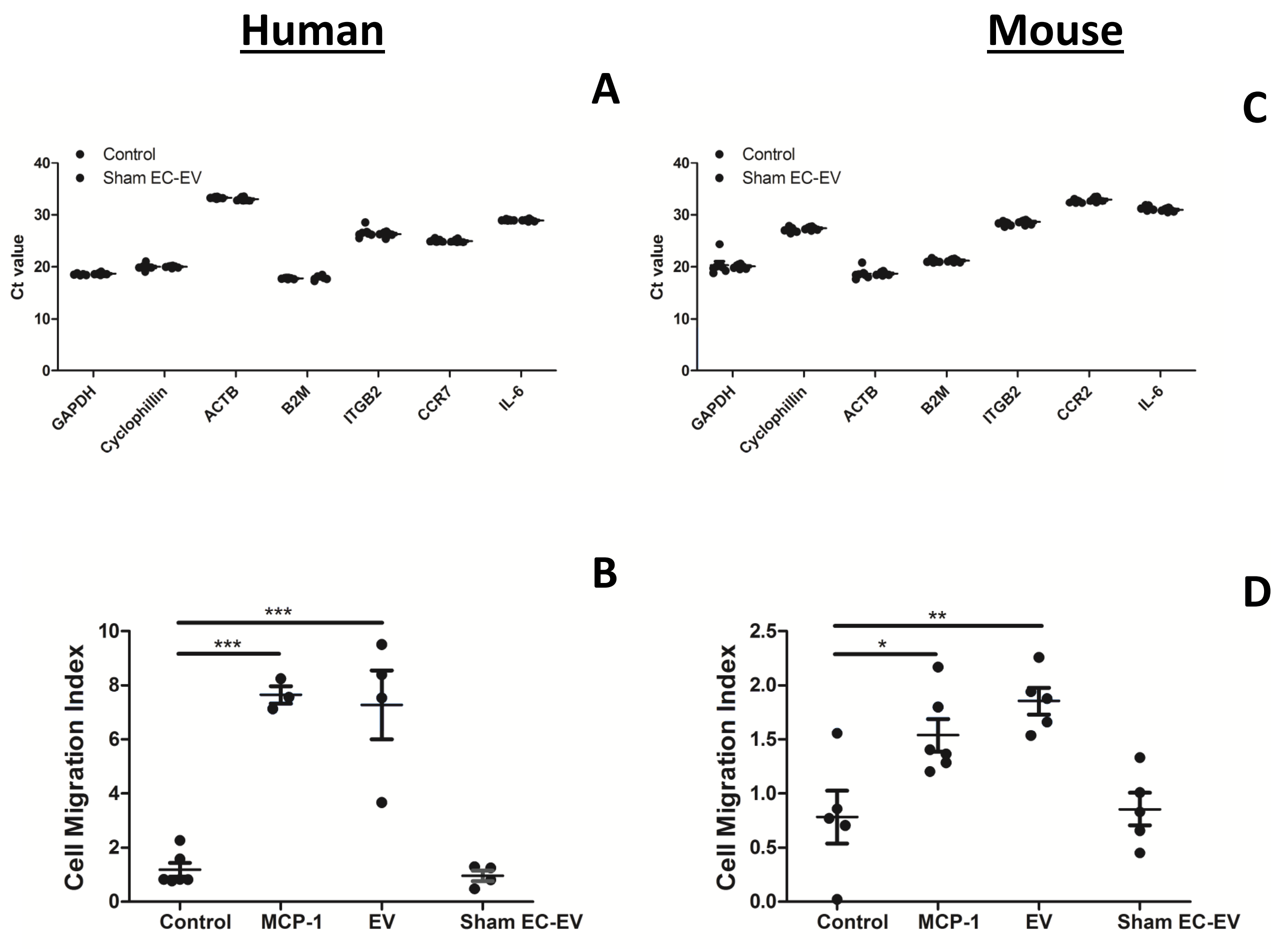

Supplementary Acknowledgments: Oxford Acute Myocardial Infarction (OxAMI) Study

Adrian Banning, Oxford University Hospitals NHS Foundation Trust

Raj Kharbanda, Oxford University Hospitals NHS Foundation Trust

Neil Ruparelia, Division of Cardiovascular Medicine, University of Oxford, UK

Mohammad Alkhalil, Division of Cardiovascular Medicine, University of Oxford, UK

Gian Liugi De Maria, Oxford University Hospitals NHS Foundation Trust

Lisa Gaughran, Division of Cardiovascular Medicine, University of Oxford, UK

Erica d'All Armellina, Division of Cardiovascular Medicine, University of Oxford, UK

Vanessa Ferreira, Division of Cardiovascular Medicine, University of Oxford, UK

Alessansdra Borlotti, Division of Cardiovascular Medicine, University of Oxford, UK
